# Incidence and factors associated of early non-response in first-treatment and drug-naïve patients with schizophrenia: a real-world study

**DOI:** 10.3389/fpsyt.2023.1173263

**Published:** 2023-04-27

**Authors:** Lin Zhang, Aohan Bai, Zhongyu Tang, Xuebing Liu, Yi Li, Jun Ma

**Affiliations:** ^1^Affiliated Wuhan Mental Health Center, Tongji Medical College of Huazhong University of Science and Technology, Wuhan, China; ^2^Department of Psychiatry, Wuhan Mental Health Center, Wuhan, China

**Keywords:** incidence, early non-response, first-treatment, drug-naïve, schizophrenia

## Abstract

**Background:**

Schizophrenia is a severe and persistent mental condition that causes disability. For subsequent clinical care, it is extremely practical to effectively differentiate between patients who respond to therapy quickly and those who do not. This study set out to document the prevalence and risk factors for patient early non-response.

**Methods:**

The current study included 143 individuals with first-treatment and drug-naïve (FTDN) schizophrenia. Patients were classified as early non-responders based on a Positive and Negative Symptom Scale (PANSS) score reduction of less than 20% after 2 weeks of treatment, otherwise as early responders. Clinical subgroups’ differences in demographic data and general clinical data were compared, and variables related to early non-response to therapy were examined.

**Results:**

Two weeks later, a total of 73 patients were described as early non-responders, with an incidence of 51.05%. The early non-response subgroup had significantly higher PANSS scores, Positive symptom subscale (PSS) scores, General psychopathology subscale (GPS) scores, Clinical global impression scale - severity of illness (CGI-SI) and Fasting blood glucose (FBG) levels compared to the early-response subgroup. CGI-SI and FBG were risk factors for early non-response.

**Conclusion:**

High rates of early non-response have been seen in FTDN schizophrenia patients, and risk variables for predicting early non-response include CGI-SI scores and FBG levels. However, we need more in-depth studies to confirm the generalizable range of these two parameters.

## 1. Introduction

Schizophrenia is a serious, chronic, highly disabling mental illness and although intensive and sustained research has been carried out, the outcomes of best practice treatment are often unsatisfactory (1). The median proportion of people with schizophrenia who met clinical and social recovery criteria was only 13.5% in a systematic review based on 50 outcome studies (2). The unemployment rate for people with schizophrenia is a staggering 89–94%, topping the list of unemployment rates for all mental illnesses (3), and their life expectancy is reduced by an average of 10–20 years (4). Despite the impressive advances in research on the pathophysiology of schizophrenia in recent years, there are still significant challenges in optimizing the treatment of the disorder (5).

Early response to treatment in schizophrenia is of great significance for better management of the disorder in the future and is touted as one of the most reliable predictors of long-term clinical outcome in schizophrenia (6). Poor early response or early non-response to treatment often strongly indicates non-response to subsequent treatment (7, 8), higher discontinuation rates (9), more severe metabolic disorder adverse reactions (10), etc. A previous review of diagnostic tests concluded that no/minimal response to antipsychotics within the first 2 weeks of treatment may be a sufficient indication for switching antipsychotics (11), rather than the 4–8 weeks of acute phase drug titration and adequate time for observation traditionally recommended by treatment guidelines (9). A multicenter randomized double-blind study demonstrated that timely switching of antipsychotic drug classes in psychiatric patients who did not respond to 2 weeks of treatment with olanzapine and amisulpride resulted in more significant improvement in psychiatric symptoms and a higher proportion of patients achieving symptom remission after 6 weeks of treatment compared to those who did not switch drugs in time (12). Consequently, the authors concluded that timely reporting of the proportion of patients who do not respond early to treatment and exploring the factors associated with obtaining early non-response at baseline level and increasing the early clinical identification of this patient group would be of great benefit in clinical work to optimize the drug management and clinical management of patients.

Although the incidence of early unresponsive patients with schizophrenia has been reported, the known reported results vary considerably due to differences in inclusion criteria and study objectives, and incidence rates from China are lacking. For example, German researchers reported an incidence of 43.43% (12), compared to 15.22% from the UK (13)and 32% from Nigeria (14). Our study is based on real clinical practice in China, reporting the incidence of patients with early non-response to treatment in daily clinical work practice, describing the clinical characteristics of this patient population, and exploring the factors associated with early non-response.

## 2. Materials and methods

### 2.1. Subjects

For the present study, 143 patients with first-treatment and drug-naïve (FTDN) schizophrenia admitted to Wuhan Mental Health Centre from May 2019 to September 2020 were selected.

Patients must meet the following inclusion criteria:

1. Meet the diagnostic criteria for schizophrenia according to the International Classification of Diseases, 10th Revision (ICD-10), and the total duration of the illness should not exceed 6 years.

2. No use of antipsychotic medication prior to the first hospitalization, or the duration of medication did not exceed 1 week.

3. The included samples were all Chinese Han.

4. Male or female, aged 18–45 years.

5. Positive and Negative Symptom Scale (PANSS) total score was more than 60 points.

Exclusion criteria: excluding breastfeeding and pregnant women, severe somatic diseases confirmed as diabetes, hypertension and diseases of the immune system; excluding bipolar disorder, intellectual disability, mental disorder due to epilepsy, major depressive disorder and other mental diseases.

The study was reviewed and approved by the Ethics Committee of Wuhan Mental Health Center, and all participants signed a written informed consent form.

### 2.2. Research design

The study, which was intended to be a real-world study, calculated the incidence of early responders to treatment in schizophrenia patients who met the inclusion criteria within a specific time frame, compared the variations in demographic data and general clinical parameters between the two clinical subgroups of early-response and early non-response, and examined the factors associated with the early-response group. Finally, we plot receiver operating characteristic (ROC) curves for the acquired correlates and determine the cut-off values that can be used to differentiate treatment response.

We have designed our own EXCLE spreadsheet for recording demographic and general clinical information on patients admitted to the hospital (Table 1). We collected and recorded patients’ first (usually on the day of admission or the next day) biochemical tests, electrocardiograms, and prescribed antipsychotic medication after admission. The clinical indicators we collected specifically included the PANSS, including its three subscales: PANSS positive score (PPS), PANSS negative score (PNS), PANSS general psychopathology scale (GPS), Clinical global impression scale - severity of illness (CGI-SI), Body weight (BW), Waist circumference (WC), Body mass index (BMI), Fasting blood glucose (FBG), Total cholesterol (TC), Triglycerides (TG), Low density lipoprotein cholesterol (LDL-C), High density lipoprotein cholesterol (HDL-C), Prolactin (PRL), QT-c interval in the electrocardiogram (QT-c), Systolic blood pressure (SBP), Diastolic blood pressure (DBP).

**Table 1:** Differences in demographic and general clinical datas between clinical subgroups.

Index	Total patients (*n* = 143)	Early-response (*n* = 70)	Early non-response (*n* = 73)	*t/χ^2^/F*	*p*
Age (years)	29.37 ± 7.30	28.22 ± 7.20	30.47 ± 7.27	−1.86	0.065
Onset age (years)	24.41 ± 6.53	23.93 ± 6.44	24.88 ± 6.62	−0.87	0.387
Course of disease (years)	2.97 ± 1.79	2.73 ± 1.93	3.21 ± 1.63	−1.60	0.112
Marital status (*n*, %)				0.82	0.366
Married	66, 46.15%	35, 50.00%	31, 42.47%		
Others^a^	77, 53.85%	35, 50.00%	42, 57.53%		
Living conditions				0.80	0.671
With spouse	62, 43.36%	32, 45.71%	30, 41.10%		
With other relatives	78, 54.55%	36, 51.43%	42, 57.53%		
Live alone	3, 2.10%	2, 2.86%	1, 1.37%		
Family history				0.06	0.810
Negative	108, 75.52%	52, 74.29%	56, 76.71%		
Positive	35, 24.48%	18, 25.71%	17, 23.29%		
Gender				3.02	0.082
Male	63, 44.06%	36, 51.43%	27, 36.99%		
Female	80, 55.94%	34, 48.57%	46, 63.01%		
Educational background				0.11	0.736
Junior school and below	92, 64.34%	46, 65.71%	46, 63.01%		
High school and above	51, 35.66%	24, 34.29%	27, 36.99%		
PANSS	89.15 ± 11.19	86.94 ± 10.20	91.27 ± 11.74	−2.35	0.020*
PSS	24.43 ± 3.94	23.53 ± 3.64	25.29 ± 4.04	−2.73	0.007*
NSS	23.29 ± 5.68	22.97 ± 5.12	23.59 ± 6.18	−0.65	0.518
GPS	41.26 ± 6.45	39.99 ± 6.17	42.48 ± 6.51	−2.35	0.020*
CGI-SI	5.36 ± 0.65	5.20 ± 0.55	5.52 ± 0.69	−3.07	0.003*
BW (kg)	58.90 ± 11.18	60.39 ± 11.05	57.48 ± 11.20	1.56	0.120
WC (cm)	78.56 ± 9.29	80.07 ± 8.58	77.11 ± 9.76	1.93	0.056
BMI (kg/m^2^)	21.65 ± 3.33	21.80 ± 3.23	21.50 ± 3.45	0.52	0.604
FBG (mmol/L)	4.52 ± 0.77	4.32 ± 0.68	4.71 ± 0.81	−3.07	0.003*
TC (mmol/L)	3.84 ± 0.58	3.83 ± 0.73	3.84 ± 0.77	−0.13	0.901
TG (mmol/L)	1.10 ± 0.99	1.13 ± 0.54	1.07 ± 0.61	0.58	0.561
LDL-C (mmol/L)	2.18 ± 0.61	2.15 ± 0.59	2.22 ± 0.62	−0.63	0.531
HDL-C (mmol/L)	1.18 ± 0.24	1.18 ± 0.25	1.18 ± 0.23	0.14	0.891
PRL (ng/mL)	18.04 ± 13.49	19.08 ± 14.78	17.03 ± 12.14	0.91	0.365
Q-Tc (ms)	399.41 ± 24.87	398.90 ± 24.78	399.90 ± 25.11	−0.24	0.81
SBP (mmHg)	113.32 ± 12.16	114.00 ± 11.94	112.67 ± 12.42	0.65	0.516
DBP (mmHg)	75.15 ± 8.77	75.83 ± 9.15	74.49 ± 8.39	0.91	0.364
Prescription drugs (*n*, %)				11.83	0.066
Aripiprazole	24, 16.78%	10, 14.29%	14, 19.18%		
Olanzapine	27, 18.88%	20, 28.57%	7, 9.59%		
Quetiapine	22, 15.38%	9, 12.86%	13, 17.81%		
Risperidone	28, 19.58%	16, 22.86%	12, 16.44%		
Ziprasidone	21, 14.69%	7, 10.00%	14, 19.18%		
Perphenazine	9, 6.29%	3, 4.29%	6, 8.22%		
Haloperidol	12, 8.39%	5, 7.14%	7, 9.59%		
Atypical antipsychotics (*n*, %)				1.16	0.281
Yes	122, 85.31%	62, 88.57%	60, 82.19%		
No	21, 14.69%	8, 11.43%	13, 17.81%		

a Others in marital status: including unmarried, divorced and widowed.

PANSS: Positive and negative symptom scale; PPS: PANSS positive score; PNS: PANSS negative score; GPS: PANSS general psychopathology scale; CGI-SI: Clinical global impression scale—severity of illness; BW: Body weight; WC: Waist circumference; BMI: Body mass index; FBG: Fasting blood glucose; TC: Total cholesterol, TG: Triglycerides, LDL-C: Low density lipoprotein cholesterol; HDL-C: High density lipoprotein cholesterol; PRL: Prolactin; Q-Tc: Q-Tc interval; SBP: Systolic blood pressure; DBP: Diastolic blood pressure. *p < 0.05.

Medication: Medication: The investigators did not interfere with the clinically prescribed medication for the patients, and the bedside doctors flexibly selected the type of antipsychotic medication for the included patients based on the patient’s particular circumstances and their individual clinical experience. The bedside physician may titrate the chosen antipsychotic drug for 2 weeks while adhering to the prescribed guidelines and taking into account the patient’s unique circumstances, but without changing the medicine.

Clinical subgroup classification rules: We distinguished between two clinical subgroups using the PANSS subtraction rate. Patients were categorized as part of the early-response group after 2 weeks of therapy if their PANSS decrease rate was more than or equal to 20%; otherwise, they were part of the early non-response group (15). The PANSS score reduction rate (%) = (baseline score – 2^nd^ weekend score) / (baseline score - 30) × 100%. The PANSS scores were also administered to the included patients by 2 professionally trained attending psychiatrists on 2 successive occasions (day of patient admission and 2 weeks after treatment). Also, the included patients received the PANSS ratings from 2 attending psychiatrists with professional training on 2 separate dates (day of patient admission and 2 weeks after treatment).

### 2.3. Data analysis

Data obtained for continuous measurements with normal distribution are expressed as means and standard deviations, and categorical variables are expressed as counts. Independent samples t-test is used to compare data from different groups. The chi-square test or Fisher’s exact probability was used to compare rates. We then constructed a binary logistic regression model with early non-response as the outcome variable and parameters that differed in the univariate analysis as independent variables to identify factors associated with the early nonresponse group. Finally, we plotted ROC curves for the factors obtained from the logistic regression that influenced early non-response, which were used to determine meaningful cut-off values. IBM SPSS (version 26.0, SPSS Inc., Chicago, IL, United States) was used for data analysis. GraphPad Prism software (version 8.4.3; GraphPad Software Inc., La Jolla, CA, USA) was used for plotting. The significance level for all statistical tests was set at *p* < 0.05 (two tails).

## 3. Results

### 3.1. Demographic and general clinical data of enrolled patients

The average doses of the seven antipsychotic drugs at the end of 2 weeks were aripiprazole (23.75 ± 4.95) mg, olanzapine (17.78 ± 3.49) mg, quetiapine (706.82 ± 67.78), risperidone (5.26 ± 0.90) mg, ziprasidone (129.09 ± 22.02) mg, perphenazine (25.33 ± 7.21) mg and haloperidol (25.83 ± 3.66) mg. 2 weeks later, a total of 70 included patients were classified in the early-response group, with a reduction rate of (37.07 ± 14.05) %, while another 73 patients were classified in the early non-response group, with a mean reduction rate of (8.77 ± 6.44) %. The percentage of the early non-response group was 51.05%. Compared to the early-response group, the early non-response group had significantly higher PANSS scores (*p* = 0.020), PSS scores (*p* = 0.007), GPS scores (*p* = 0.020), CGI-SI scores (*p* = 0.003), and FBG (*p* = 0.003).

### 3.2. Factors influencing for early-response in included patients

Next, we focused on the related factors of early-response group. A binary logistic regression model (backward: Wald) was constructed with early non-responder as the outcome variable and parameters with differences in univariate analysis as independent variables. As shown in Table 2, CGI-SI (*B* = 0.68, *p* = 0.020, OR = 1.98), and FBG (*B* = 0.65, *p* = 0.021, OR = 1.91) were risk factors for early non-response.

**Table 2:** Factors influencing early non-response in all included patients: a binary logistic regression model.

	Coefficients	Std. error	Wald	*p* value	95% CI for EXP (B)		
	B				Exp(B)	Lower	Upper
Constant	−6.52	1.89	11.90	0.001	0.00		
CGI-SI	0.68	0.29	5.37	0.020*	1.98	1.11	3.52
FBG	0.65	0.28	5.35	0.021*	1.91	1.10	3.29

CGI-SI: Clinical global impression scale—severity of illness; FBG: Fasting blood glucose; *p < 0.05.

### 3.3. ROC curve analysis to distinguish early non-responder from early responder

**Figure 1:**
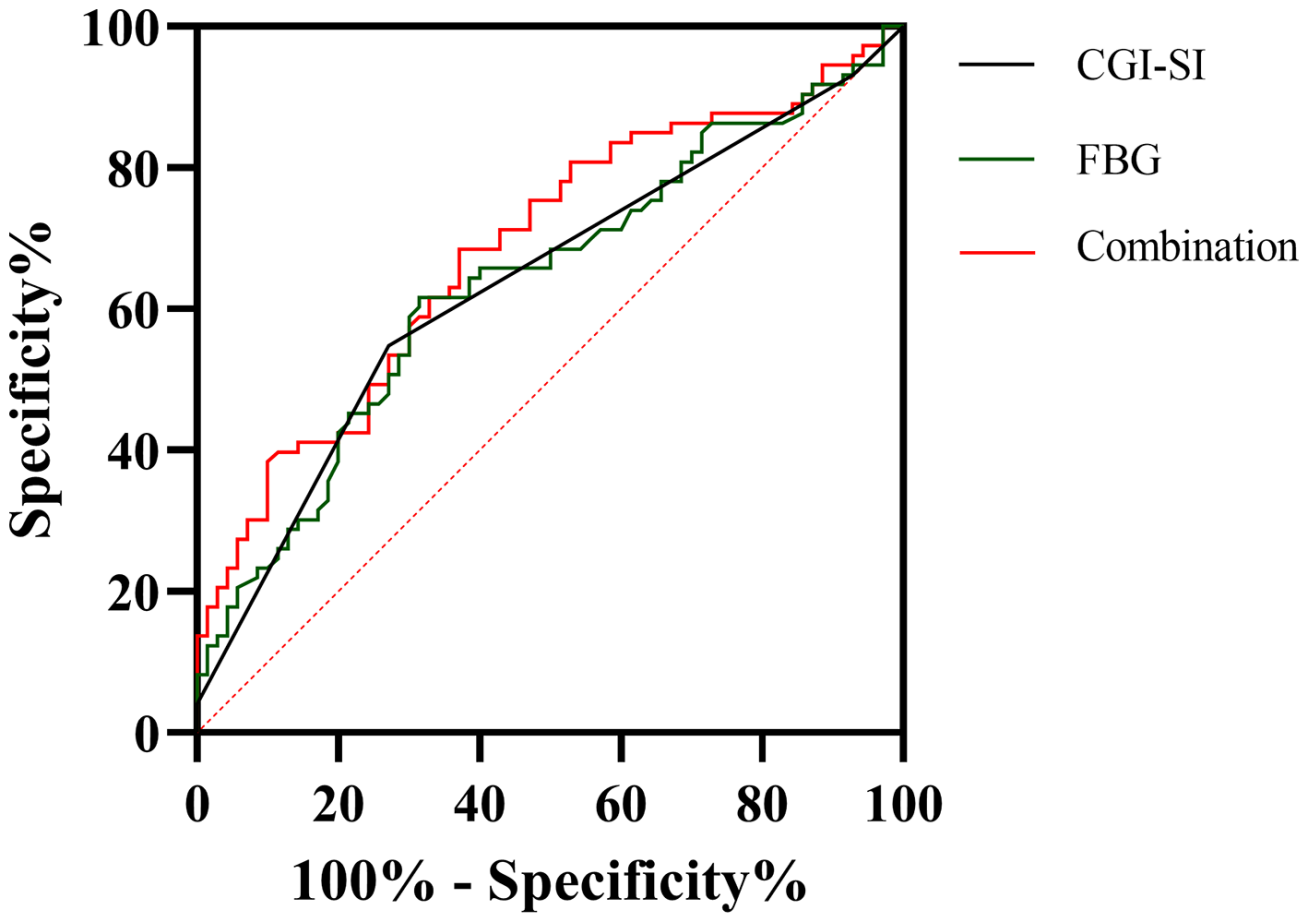
The discriminatory capacity of related factors for between early non-response and early response in including patients. The area under the curve for CGI-SI and FBG is 0.635 and 0.637, respectively.

Finally, we plotted the ROC curves for both CGI-SI and FBG variables, as shown in Figure 1. AUCROC showed the following values for the two factors: CGI-SI was 0.635 (*p* = 0.005, 95% CI = 0.544–0.726) and FBG was 0.637 (*p* = 0.005, 95% CI = 0.546–0.728). According to the Youden index, the optimal cut-off value for CGI-SI was 6 (Youden index = 0.28, sensitivity = 55%, specificity = 73%) and the optimal cut-off value for FBG was 4.53 mmol/l (Youden index = 0.30, sensitivity = 62%, specificity = 69%).

## 4. Discussion

We provide genuine information on the clinical incidence and variables linked to early non-response in FTDN schizophrenia patients as part of a real-world clinical investigation. The following are the study’s primary conclusions: 1. The PANSS scores (both PSS and GPS scores), CGI-SI scores, and fasting blood glucose levels were all higher in the clinical subgroup of early non-response with FTDN schizophrenia than they were in the clinical subgroup of early-response. 2. The early non-response was predicted by the CGI-SI scores and FBG levels. 3.The optimal cut-off values for the two parameters of 6 for CGI-SI and 4.53 mmol/l for FBG.

We discovered that the severity of psychopathology was higher in FTDN schizophrenia patients categorized as early non-responders after 2 weeks of treatment. Two clinical studies, which strictly limited prescribed medications, reported differing results from ours, finding that the early non-response subgroup had significantly higher disease severity at baseline (based on total PANSS scores rather than CGI-SI) in first-episode schizophrenia patients (9, 16). However, two other studies reported findings highly consistent with ours; specifically, early non-responders exhibited more severe psychopathological symptoms, including higher PANSS total scores and its three subscale scores (17, 18). The contradictory results may stem from strict restrictions on prescribed medications and enrollment criteria. Furthermore, research has indicated that metabolic disturbances, such as BW (19) and FBG (10), are more severe in treatment non-responders even before antipsychotics are prescribed. In light of our findings, it is reasonable to assert that FTDN schizophrenia patients with poor treatment response not only suffer from more severe psychiatric symptoms but also experience more pronounced metabolic disturbances.

The available known studies that focus on the incidence of early non-response to treatment for schizophrenia report a high degree of heterogeneity, and almost without exception these have been conducted under the premise of setting strict antipsychotic drug classes as inclusion criteria, with few real-world studies involved. A clinical trial from South Africa prescribing long-acting flupenthixol injections to patients with first-episode schizophrenia reported a 12% (15/126) incidence of treatment non-response (16). Another clinical trial, which did not strictly limit the duration of included cases, reported a 72.4% incidence of early non-response in patients with schizophrenia spectrum disorders after 2 weeks of risperidone treatment in the acute phase (20). There also is a multicenter randomized controlled clinical study reporting a 35% incidence of early non-response in adolescent schizophrenia patients prescribed olanzapine (21). As a real-world clinical study, we did not restrict the type of medication prescribed to the patients enrolled *a priori*, and we reported a 51.05% incidence of early non-response, more than half of the total sample, and reported differently in all the above studies.

In addition to the impact of differences in study design and enrolment criteria on response rates, the diversity of instruments and assessment methods used to assess response rates is one of the key influences on the heterogeneity of response rates. For example, a large German study reported early non-response rates in 528 severely impaired schizophrenic patients using four different assessment tools, respectively, an incidence of 30% reported using the PANSS positive score, 41% reported using the PANSS negative score, and the CGI-severity score reported at 63% and Scale of Occupational and Functional Assessment (SOFAS) reported at 35% (22). In parallel to the above-mentioned tools for differentiating non-responder from early responder, there are other assessment tools that are considered to have the same efficacy (23). In addition, the time cut-offs involved in some of the studies to differentiate between early-response and non-response varied. For example, a time node of 3 weeks of treatment was used (24), as well as a time node of 2 weeks (15, 20), and one study even pushed for a 1 week time scenario (25). The present study used a PANSS reduction rate of greater than or equal to 20% after 2 weeks of treatment as the cut-off point for treatment response or non-response. In summary, whatever the assessment tool used and whatever the time cut-off point, any effective separation of early non-responders from the patient population is to be acknowledged. A realistic implication of this is to implement targeted clinical management measures as soon as possible to improve the prognosis and adverse effects in this subgroup of patients (10).

As with incidence, there are similarly no more consistent and universally accepted conclusions about the predictors of early non-response to treatment for schizophrenia. There are many factors that influence the prognosis and outcome of people with first-episode schizophrenia, such as duration of untreated after the episode (26, 27), degree of weight gain after treatment (28), levels of self-awareness (29) and the presence or absence of certain specific genetic variants (30). However, none of these predictors were specifically developed to predict whether a patient was an early responder. A small sample of clinical studies restricted to three atypical antipsychotics found no valid factors, including drug type and drug dose, that could be used to predict early response in patients with relapsing schizophrenia (8). Another small multicenter clinical study from Japan found greater improvement in patients prescribed risperidone within 2 weeks of the acute phase and that the Clinical Global Impressions-improvement scale (CGI-I) rating at the end of the 2 week could be used for the long-term outcome of patients prescribed risperidone for initial treatment, but could not be generalized to patients on olanzapine (31). A further study that included 135 patients with schizophrenia in the acute phase who were prescribed levotepine found that a reduction of 6 points on the Brief Psychiatric Rating Scale (BPRS) positive symptom subscale at the end of 2 weeks of treatment served as the best predictor of treatment response (32). In summary, previous studies have focused on exploring or optimizing the best predictors of patient response after drug treatment (e.g., 2 weeks after treatment), and the indicators reported for prediction have been inconsistent. These are certainly distinct from our study’s attempts to explore the ideas that can be used to predict factors associated with treatment response at baseline. Our study found that CGI-SI scores and fasting glucose levels at baseline are relevant predictors of treatment non-response in weekend 2 patients.

Finally, we finally plotted risk factor ROC curves and reported optimal cut-off values for two parameters of 6 for CGI-SI and 4.53 mmol/l for FBG, respectively. A limited number of studies, using Global Assessment of Functioning (GAF) as the target variable, found that baseline GAF score (*c*-statistics = 0.85, 95% CI 0.77, 0.93; boostrap estimate 0.8598) is a predictor of good response to treatment at 6 months (33). In terms of specific statistical parameters, this prediction model appears to be superior to the model we reported. Unfortunately, the investigators did not report the optimal cut-off value. In addition, several studies have explored the use of brain imaging and genomics protocols to identify and predict early treatment response in schizophrenia (34–36). However, it is still inconclusive and the generalizability of our reported optimal cut-off values for the two parameters needs to be further determined by prospective studies.

The current study has a few other flaws as well. The lack of sample size continues to be a significant problem restricting the statistical effectiveness of this investigation, which may adversely affect the in-depth generalization of the statistical results and the conclusions despite the fact that it is a real-world study. Moreover, baseline assessments may not include clinical indications like self-awareness and cognitive function that may affect therapy response. Finally, we have not addressed biological signs that may be more recognizable and predictively important.

In conclusion, the incidence of early non-response in FTDN schizophrenia patients is understandably high, and we identified the following risk factors: CGI-SI scores and FBG levels. Studies with larger sample sizes are needed to further identify potential risk factors to increase the identification of patients with early non-response to improve patient outcomes.

## Data availability statement

The raw data supporting the conclusions of this article will be made available by the authors, without undue reservation.

## Author contributions

JM and YL made substantial contributions to conception and design of the review and gave final approval of the version to be published. LZ drafted the manuscript. AB and ZT revised the manuscript critically for important intellectual content. XL ensured that questions related to the accuracy or integrity of any part of the work were appropriately investigated and resolved. All authors contributed to the article and approved the submitted version.

## Funding

This study was funded by the scientific research project of Wuhan Municipal Health Commission (WG20D12, ZT, PI).

## Conflict of interest

The authors declare that the research was conducted in the absence of any commercial or financial relationships that could be construed as a potential conflict of interest.

## Publisher’s note

All claims expressed in this article are solely those of the authors and do not necessarily represent those of their affiliated organizations, or those of the publisher, the editors and the reviewers. Any product that may be evaluated in this article, or claim that may be made by its manufacturer, is not guaranteed or endorsed by the publisher.

## References

[1] Charlson FJ Ferrari AJ Santomauro DF Diminic S Stockings E Scott JG. Global epidemiology and burden of schizophrenia: findings from the global burden of disease study 2016. Schizophr Bull. (2018) 44:1195–203. doi: 10.1093/schbul/sby058, PMID: 29762765 PMC6192504

[2] Jääskeläinen E Juola P Hirvonen N McGrath JJ Saha S Isohanni M. A systematic review and meta-analysis of recovery in schizophrenia. Schizophr Bull. (2013) 39:1296–306. doi: 10.1093/schbul/sbs130, PMID: 23172003 PMC3796077

[3] Hakulinen C Elovainio M Arffman M Lumme S Pirkola S Keskimäki I. Mental disorders and long-term labour market outcomes: Nationwide cohort study of 2 055 720 individuals. Acta Psychiatr Scand. (2019) 140:371–81. doi: 10.1111/acps.13067 31254386

[4] Chesney E Goodwin GM Fazel S. Risks of all-cause and suicide mortality in mental disorders: a meta-review. World Psychiatry. (2014) 13:153–60. doi: 10.1002/wps.20128, PMID: 24890068 PMC4102288

[5] Owen MJ Sawa A Mortensen PB. Schizophrenia. Lancet. (2016) 388:86–97. doi: 10.1016/s0140-6736(15)01121-6 26777917 PMC4940219

[6] Schennach-Wolff R Seemüller FH Mayr A Maier W Klingberg S Heuser I. An early improvement threshold to predict response and remission in first-episode schizophrenia. Br J Psychiatry. (2010) 196:460–6. doi: 10.1192/bjp.bp.109.069328 20513856

[7] Stauffer VL Case M Kinon BJ Conley R Ascher-Svanum H Kollack-Walker S. Early response to antipsychotic therapy as a clinical marker of subsequent response in the treatment of patients with first-episode psychosis. Psychiatry Res. (2011) 187:42–8. doi: 10.1016/j.psychres.2010.11.017 21168920

[8] Chen YL Chen KP Chiu CC Tai MH Lung FW. Early predictors of poor treatment response in patients with schizophrenia treated with atypical antipsychotics. BMC Psychiatry. (2018) 18:376. doi: 10.1186/s12888-018-1950-1, PMID: 30509308 PMC6278161

[9] Kinon BJ Chen L Ascher-Svanum H Stauffer VL Kollack-Walker S Sniadecki JL. Predicting response to atypical antipsychotics based on early response in the treatment of schizophrenia. Schizophr Res. (2008) 102:230–40. doi: 10.1016/j.schres.2008.02.021 18423985

[10] Zhu J Wu J Liu X Ma J. Relationship between efficacy and common metabolic parameters in first-treatment drug-Naïve patients with early non-response schizophrenia: a retrospective study. Ann General Psychiatry. (2023) 22:6. doi: 10.1186/s12991-023-00436-3, PMID: 36800967 PMC9936715

[11] Samara MT Leucht C Leeflang MM Anghelescu IG Chung YC Crespo-Facorro B. Early improvement as a predictor of later response to antipsychotics in schizophrenia: a diagnostic test review. Am J Psychiatry. (2015) 172:617–29. doi: 10.1176/appi.ajp.2015.14101329 26046338

[12] Heres S Cordes J Feyerabend S Schmidt-Kraepelin C Musil R Riedel M. Changing the antipsychotic in early nonimprovers to Amisulpride or olanzapine: randomized, double-blind trial in patients with schizophrenia. Schizophr Bull. (2022) 48:1273–83. doi: 10.1093/schbul/sbac068, PMID: 35857811 PMC9673269

[13] Millgate E Griffiths K Egerton A Kravariti E Casetta C Deakin B. Cognitive function and treatment response trajectories in first-episode schizophrenia: evidence from a prospective cohort study. BMJ Open. (2022) 12:e062570. doi: 10.1136/bmjopen-2022-062570, PMID: 36410817 PMC9680154

[14] Ezeme MS Uwakwe R Ndukuba AC Igwe MN Odinka PC Amadi K. Clinical correlates of treatment response among patients with schizophrenia in a tertiary Nigerian hospital. J Health Care Poor Underserved. (2017) 28:721–38. doi: 10.1353/hpu.2017.0070, PMID: 28529220

[15] Loebel A Citrome L Correll CU Xu J Cucchiaro J Kane JM. Treatment of early non-response in patients with schizophrenia: assessing the efficacy of antipsychotic dose escalation. BMC Psychiatry. (2015) 15:271. doi: 10.1186/s12888-015-0629-0, PMID: 26521019 PMC4628370

[16] Chiliza B Asmal L Kilian S Phahladira L Emsley R. Rate and predictors of non-response to first-line antipsychotic treatment in first-episode schizophrenia. Hum Psychopharmacol. (2015) 30:173–82. Epub 20150311. doi: 10.1002/hup.2469 25758549

[17] Schennach-Wolff R Jäger M Mayr A Meyer S Kühn KU Klingberg S. Predictors of response and remission in the acute treatment of first-episode schizophrenia patients--is it all about early response? Eur Neuropsychopharmacol. (2011) 21:370–8. doi: 10.1016/j.euroneuro.2010.10.003 21255982

[18] Wold KF Ottesen A Camilla BF Johnsen E Lagerberg TV Romm KL. Early identification of treatment non-response in first-episode psychosis. Eur Psychiatry. (2023) 66:e30. doi: 10.1192/j.eurpsy.2023.15 36915260 PMC10134449

[19] Chen L Ascher-Svanum H Stauffer V Kinon BJ Kollack-Walker S Ruberg S. Optimal thresholds of early response to atypical antipsychotics: application of signal detection methods. Schizophr Res. (2009) 113:34–40. doi: 10.1016/j.schres.2009.06.001 19564097

[20] Kinon BJ Chen L Ascher-Svanum H Stauffer VL Kollack-Walker S Zhou W. Early response to antipsychotic drug therapy as a clinical marker of subsequent response in the treatment of schizophrenia. Neuropsychopharmacology. (2010) 35:581–90. doi: 10.1038/npp.2009.164, PMID: 19890258 PMC3055392

[21] Stentebjerg-Olesen M Ganocy SJ Findling RL Chang K DelBello MP Kane JM. Early response or nonresponse at week 2 and week 3 predict ultimate response or nonresponse in adolescents with schizophrenia treated with olanzapine: results from a 6-week randomized, placebo-controlled trial. Eur Child Adolesc Psychiatry. (2015) 24:1485–96. Epub 20150602. doi: 10.1007/s00787-015-0725-1 26032132

[22] Lambert M Schimmelmann BG Naber D Eich FX Schulz H Huber CG. Early- and delayed antipsychotic response and prediction of outcome in 528 severely impaired patients with schizophrenia treated with Amisulpride. Pharmacopsychiatry. (2009) 42:277–83. doi: 10.1055/s-0029-1234105 19924588

[23] Lin CH Lin HS Lin SC Kuo CC Wang FC Huang YH. Early improvement in Panss-30, Panss-8, and Panss-6 scores predicts ultimate response and remission during acute treatment of schizophrenia. Acta Psychiatr Scand. (2018) 137:98–108. doi: 10.1111/acps.12849 29280500

[24] Hatta K Otachi T Sudo Y Kuga H Takebayashi H Hayashi H. A comparison between augmentation with olanzapine and increased risperidone dose in acute schizophrenia patients showing early non-response to risperidone. Psychiatry Res. (2012) 198:194–201. doi: 10.1016/j.psychres.2012.01.006 22421064

[25] O'Gorman C Kapur S Kolluri S Kane J. Early improvement on antipsychotic treatment as a predictor of subsequent response in schizophrenia: analyses from ziprasidone clinical studies. Hum Psychopharmacol. (2011) 26:282–90. doi: 10.1002/hup.1200 21638329

[26] Perkins DO Gu H Boteva K Lieberman JA. Relationship between duration of untreated psychosis and outcome in first-episode schizophrenia: a critical review and meta-analysis. Am J Psychiatry. (2005) 162:1785–804. doi: 10.1176/appi.ajp.162.10.1785, PMID: 16199825

[27] Murru A Carpiniello B. Duration of untreated illness as a key to early intervention in schizophrenia: a review. Neurosci Lett. (2018) 669:59–67. doi: 10.1016/j.neulet.2016.10.003, PMID: 27717830

[28] Chen YQ Li XR Zhang L Zhu WB Wu YQ Guan XN. Therapeutic response is associated with antipsychotic-induced weight gain in drug-naive first-episode patients with schizophrenia: an 8-week prospective study. J Clin Psychiatry. (2021) 82:20m13469. doi: 10.4088/JCP.20m13469, PMID: 34004092

[29] Ramu N Kolliakou A Sanyal J Patel R Stewart R. Recorded poor insight as a predictor of service use outcomes: cohort study of patients with first-episode psychosis in a large mental healthcare database. BMJ Open. (2019) 9:e028929. doi: 10.1136/bmjopen-2019-028929, PMID: 31196905 PMC6577359

[30] Drögemöller BI Emsley R Chiliza B van der Merwe L Wright GE Daya M. The identification of novel genetic variants associated with antipsychotic treatment response outcomes in first-episode schizophrenia patients. Pharmacogenet Genomics. (2016) 26:235–42. doi: 10.1097/fpc.0000000000000213, PMID: 26928376

[31] Hatta K Otachi T Sudo Y Hayakawa T Ashizawa Y Takebayashi H. Difference in early prediction of antipsychotic non-response between risperidone and olanzapine in the treatment of acute-phase schizophrenia. Schizophr Res. (2011) 128:127–35. doi: 10.1016/j.schres.2011.02.011 21420283

[32] Lin CH Chou LS Lin CH Hsu CY Chen YS Lane HY. Early prediction of clinical response in schizophrenia patients receiving the atypical antipsychotic Zotepine. J Clin Psychiatry. (2007) 68:1522–7. doi: 10.4088/jcp.v68n1008, PMID: 17960966

[33] Agid O Siu CO Pappadopulos E Vanderburg D Remington G. Early prediction of clinical and functional outcome in schizophrenia. Eur Neuropsychopharmacol. (2013) 23:842–51. doi: 10.1016/j.euroneuro.2012.10.005 23141372

[34] Cui LB Zhang YJ Lu HL Liu L Zhang HJ Fu YF. Thalamus Radiomics-based disease identification and prediction of early treatment response for schizophrenia. Front Neurosci. (2021) 15:682777. doi: 10.3389/fnins.2021.682777, PMID: 34290581 PMC8289251

[35] Cui LB Fu YF Liu L Wu XS Xi YB Wang HN. Baseline structural and functional magnetic resonance imaging predicts early treatment response in schizophrenia with Radiomics strategy. Eur J Neurosci. (2021) 53:1961–75. Epub 20201224. doi: 10.1111/ejn.15046, PMID: 33206423

[36] Zong X He C Huang X Xiao J Li L Li M. Predictive biomarkers for antipsychotic treatment response in early phase of schizophrenia: multi-Omic measures linking subcortical covariant network, transcriptomic signatures, and peripheral epigenetics. Front Neurosci. (2022) 16:853186. doi: 10.3389/fnins.2022.853186, PMID: 35615285 PMC9125083

